# The surgical management of non-malignant aerodigestive fistula

**DOI:** 10.1186/s13019-018-0799-1

**Published:** 2018-11-15

**Authors:** Yassar A. Qureshi, M. Muntzer Mughal, Sheraz R. Markar, Borzoueh Mohammadi, Jeremy George, Martin Hayward, David Lawrence

**Affiliations:** 1 Department of Oesophago-Gastric Surgery, University College London Hospital, 250 Euston Road, London, NW1 2BU UK; 2 Department of Surgery and Cancer, Imperial College London, London, UK; 3 Department of Thoracic Medicine, University College London Hospital, London, UK; 4 Department of Thoracic Surgery, University College London Hospital, London, UK

**Keywords:** Aerodigestive fistula, Tracheo-oesophageal fistula, Oesophageal cancer, Oesophageal surgery

## Abstract

**Background:**

Acquired aerodigestive fistula (ADF) are rare, but associated with significant morbidity. Surgery affords the best prospect of cure. We present our experience of the surgical management of ADFs at a specialist unit, highlighting operative techniques, challenges and assess clinical outcomes following intervention. We also illustrate findings of a Hospital Episodes Statistics search for ADFs.

**Methods:**

A prospectively-maintained database was searched to identify all patients diagnosed with an ADF who were managed at our institution. Of 48 patients with an ADF, eight underwent surgical intervention.

**Results:**

Four patients underwent an exploration of the ADF with primary repair of the defect. Two of these patients had proximal ADFs, amenable to repair through a neck incision, and two required a thoracotomy. Two patients suffered fistulae secondary to endoscopic therapy and underwent oesophageal exclusion surgery, with subsequent staged reconstruction. Two patients with previous Tuberculosis had a lung segmentectomy and lobectomy respectively, and a further patient in remission after treatment for lymphoma underwent oesophageal resection with synchronous reconstruction. Three patients suffered a complication, with one post-operative mortality. The remaining seven patients all achieved normal oral alimentation, with no evidence of ADF recurrence at a median follow-up of 32 months.

**Conclusions:**

Surgery to manage ADFs is effective in restoring normal alimentation and alleviates soiling of the airway, with a very low risk of recurrence. Several operative techniques can be utilised dependent on the features of the ADF. Early referral to specialist units is advocated, where the expertise to facilitate the complete management of patients is present, within a multi-disciplinary setting.

## Background

Surgical intervention affords the best prospect of long-term cure of aerodigestive fistulae (ADF). Although several operative techniques can be used to treat this debilitating condition, they can only be utilised in selected patients owing to both the underlying diagnosis and the risks associated with such surgery [1–4]. However, with ADFs becoming an increasing health problem, with improving diagnosis and evolving peri-operative care, it is likely that surgery will play a more important role in the management of ADFs.

The choice of operative technique to treat ADFs is dependent on several factors. However, the most important facet relates to the underlying oesophageal or airway disease, which determines the state of tissue and its amenability to repair and future surveillance, if required [1, 2, 5, 6]. Patients often present in a physiologically challenged state owing to the nature of the disease, and many will not be candidates for surgery. However, focused pre-operative intervention and nutritional support may enable some patients to proceed to surgery. For these reasons, a multi-disciplinary (MDT) approach is necessary, and underscores why these patients should be managed in dedicated centralised units. The range of operations include resection and reconstruction, exclusion and bypass of the affected segment of oesophagus, and exploration and repair of the ADF. The expertise of head and neck, thoracic and oesophago-gastric surgeons is required to manage these patients.

In this study, we present our experience of surgical intervention in patients diagnosed with an ADF. We explore the background leading to the development of an ADF, and relate how this can impact on the nature of surgery performed. Furthermore, we describe the operative technique, challenges and outcomes following intervention. We review the pertinent literature to enable an evidence-based approach to the surgical management of ADFs. We also illustrate findings of a Hospital Episodes Statistics (HES) search for ADFs, highlighting the challenges of diagnosis, management and reporting in contemporary practise.

## Methods

We interrogated a prospectively-maintained database to identify patients diagnosed with an ADF and managed at our institution between January 2005 and January 2017. A total of 48 patients with an ADF were identified, of whom eight patients have undergone surgery to treat their fistula. All patients were discussed at a specialist MDT where a consensus on optimal management was reached. Of the 40 patients managed non-surgically, 31 were treated with endoscopic intervention (oesophageal or tracheal stent), mostly owing to the presence of advanced malignancy not amenable to curative treatment. Endoscopic treatment facilitated an alleviation of respiratory soiling, whilst allowing oncological treatment to be commenced. A further seven patients were managed in palliative setting after presenting *in extremis*, and two patients with very small asymptomatic ADFs were managed conservatively with regular surveillance. Follow-up refers to time from diagnosis of ADF (or underlying disease where specified) to last clinical engagement or death. Median follow-up was 32 months. Local ethical approval for retrieval and use of clinical data for this study was granted.

### Operative technique

When considering surgery as treatment for ADF, several factors should be specifically assessed for. It is imperative that a careful search for malignancy is performed prior to surgery, particularly as many patients will have a preceding history of proximal oesophageal squamous cell carcinoma (SCC) treated with chemo-radiotherapy. If active malignancy is present in the context of an ADF, this represents locally advanced disease with poor outcome, rarely amenable to curative surgical intervention. In these patients, endocopic treatment should be considered to alleviate symptoms, coupled with chemo-radiotherapy if appropriate. The physiological state of the patient must also be thoroughly assessed, to ensure that the risks of major morbidity and mortality after surgery are minimised, and that the patient would be able to recover from such intervention. Patients should be carefully optimised, and where indicated, the pre-operative placement of a feeding jejunostomy and a venting gastrostomy to improve the nutritional and metabolic state, and to minimise continued soiling of the airway, should be performed.

Once a patient is deemed to have an ADF curable by surgery, secondary factors relating to the ADF and surrounding tissue become important considerations. A larger defect, a history of previous local radiotherapy and endoscopic intervention are all factors which make surgery more challenging. Also, the location of the ADF is important, as more proximally sited fistulae are amenable to repair through a neck incision, yet for distal ADF a thoracotomy is mandated, carrying a greater risk of major morbidity and mortality. If there has been significant local contamination, then it may be prudent not perform a synchronous reconstruction, as the likelihood of an anastomotic dehiscence increases. In these patients, a delayed reconstruction confers improved chances of better recovery. However, given the heterogenous aetiology of ADF, each case should be considered with a view to an individualised treatment plan.

At induction, for tracheo-oesophageal fistulae (TOF), it is important that the endotracheal tube balloon is sited distal to the fistula. This will avoid inadvertent damage to the cuff whilst dissecting and exposing the fistula, and negate the possibility of ventilatory embarrassment intra-operatively. Furthermore, this manoeuvre minimises further contamination of the respiratory tract by manipulation of the affected structures during surgery.

### ADF exploration and repair

This may involve either an incision in the neck for proximal fistulae, or a thoracotomy for more distal ADFs. In the neck, dissection must proceed to mobilise the thyroid with careful identification and preservation of the recurrent laryngeal nerves and parathyroid glands. The oesophagus should be circumferentially mobilised, as this manoeuvre will allow the pharyngo-laryngeal complex to be gently pulled superiorly and away from the thoracic inlet, to provide good access to the fistula. Once the fistula has been identified, it can be dissected free and a primary repair of the oesophagus and trachea with absorbable sutures can be performed. It is critical that the fistula is accessible from both sides of the neck to ensure complete control of the airway during the repair, whilst also facilitating a pedicled strap muscle interposition flap. This reinforces the repair by providing a physical barrier between the two suture lines.

In the thorax, a similar approach is used with an intercostal flap which is carefully prepared at the time of thoracotomy. Once the fistula has been identified, again, it is dissected free and a primary repair performed, with the intercostal flap placed between the suture lines.

### Exclusion

Exclusion surgery involves isolating the oesophagus from alimentary tract continuity, both proximal and distal to the fistula. This involves an incision in the neck to access the proximal oesophagus, where, once circumferentially mobilised, it is transected above the fistula and brought to the skin as an oesophagostomy. If the fistula is very proximal, then the superior oesophagus may be left in situ, and a large T-tube placed in the lumen with the distal limb of the tube brought to the skin.

Next, a laparotomy is performed where the oesohago-gastric junction (OGJ) is mobilised and the stomach transected below this, from the lesser curve through to the fundus. This manoeuvre excludes the oesophagus from the GI tract entirely, whilst preserving the majority of the stomach for future reconstruction. The small stomach remnant attached to the OGJ is brought to the abdominal wall, where a generous gastrostomy is fashioned. This allows retrograde access to the excluded oesophagus, for both endoscopic surveillance and therapy, and facilitates venting of oesophageal mucous.

Our unit policy is to defer reconstruction as a second, staged procedure. This allows the patient a period of recovery, whilst respiratory and nutritional optimisation continues. Furthermore, by fashioning an anastomosis at the index operation in a potentially contaminated surgical field, there is a higher chance of a leak. If this were to occur, there is substantial risk of fistula recurrence. Where possible, the stomach is used a conduit, and is brought to the neck through the retrosternal space, thus avoiding the need for a repeat thoracotomy. If there is insufficient proximal oesophagus, the stomach may be anastomosed directly to the inferior pharyngeal constrictors.

### Resection

This is normally reserved for large or recurrent fistulae. For proximally sited ADF - those affecting the trachea, this will involve resection of the oesophagus, via a transthoracic approach. The fistula is identified, and the oesophagus dissected away around it. However, the oesophageal tissue intimately involved with the fistula is left in situ, thus avoiding direct dissection of the trachea and minimising the risk of an air leak. The tracheal defect with the overlying oesophageal tissue is then primarily closed, with the latter acting as a buttress reinforcing the tracheal repair. Typically, a gastric conduit is utilised for reconstruction, necessitating a laparotomy.

For more distal ADF, those affecting the bronchus intermedius and more distal, a thoracotomy is performed to identify the fistula. A segmentectomy or lobectomy of the lung can be performed, dependent on the size of the defect and the quality of the surrounding parenchyma. Thus, the affected distal airway and the fistula are excised *en bloc*. The oesophageal defect can be repaired primarily, utilising an intercostal flap to reinforce the repair, or an oesophageal resection is performed if the defect is very large and unlikely to heal. In these instances, given the anastomosis will be at a distinct site from the ADF, a synchronous reconstruction can be performed safely.

In our experience, tracheal resection is a very challenging operation, with the risk of significant short and long-term complications [2, 3]. Owing to the limited vascularity of the trachea, healing, particularly in this cohort of patients, may be protracted, necessitating prolonged mechanical ventilation. Thus, we have preferred to avoid such an operative intervention. However, for very large TOFs, or those where a circumferential injury to the trachea is present (such as cuff related fistulae), or where other intervention has failed, tracheal resection and reconstruction may be indicated. Mathisen et al provide an operative description and experience of this technique [3].

## Results

### Preceding history and previous intervention

**Fig. 1:**
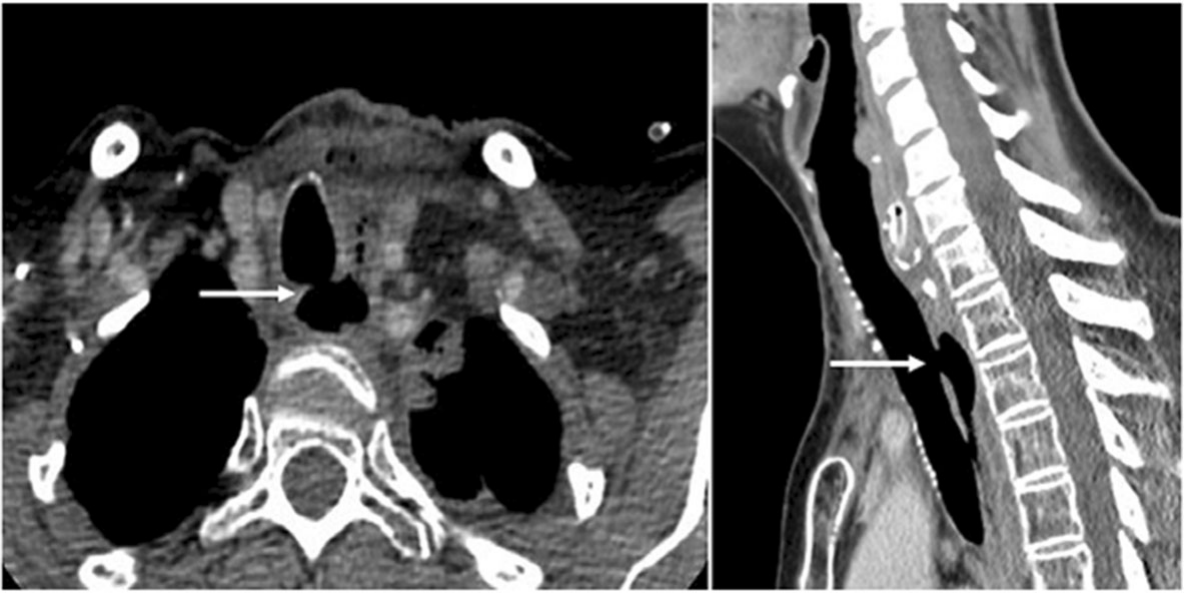
CT scan of Patient 1 demonstrating the aerodigestive fistula (arrows)

The median age at diagnosis of ADF was 56 years (range 29–73 years). Three patients had a previous diagnosis of oesophageal malignancy; all were treated with chemo- and/or radiotherapy, and one with surgical resection in addition. Two patients had a prior diagnosis of Tuberculosis (TB) and had received anti-microbial therapy in the past, and one patient previously had surgical intervention following a post-emetic oesophageal leak. In two patients, no obvious cause of ADF was identified, likely representing congenital fistulae that had persisted into adulthood. Of these cases, two patients (1 and 6) developed oesophageal strictures after their initial treatment. Patient 1 had undergone several balloon dilations and stent placements, with the stent subsequently eroding into the airway (Fig. 1). Similarly, patient 6 also received a stent which directly caused the fistula. Table 1 summarises key patient factors.

**Table 1:** Preceding history and intervention, prior to the diagnosis of ADF

Patient	Age at ADF Diagnosis (years)	Sex	Preceding Diagnosis	Preceding Intervention	Chemo-Radiotherapy	Preceding Treatment Related Complication	Previous Endotherapy
1	60	F	SSC^a^ Proximal Oesophagus	Definitive chemo-radiotherapy	Chemo-radiotherapy	Radiotherapy related stricture	-×2 stents -×3 dilations
2	57	M	TB^b^	Medical therapy	–	–	–
3	59	M	Adenocarcinoma Distal Oesophagus	Ivor-Lewis Oesophagectomy	Neo-adjuvant chemotherapy	Anastomotic leak	–
4	73	M	Unknown	–	–	–	–
5	32	F	TB^b^	Medical Therapy	–	–	–
6	33	M	Boerhaave’s Syndrome	Repair of leak	–	Re-leak; stricture	-× 1 stent
7	55	F	Oesophageal B Cell lymphoma	Chemotherapy	Chemotherapy	–	–
8	29	F	Unknown	–	–	–	–

^a^SCC- Squamous Cell Carcinoma; ^b^ TB- Tuberculosis

### ADF characteristics

**Fig. 2:**
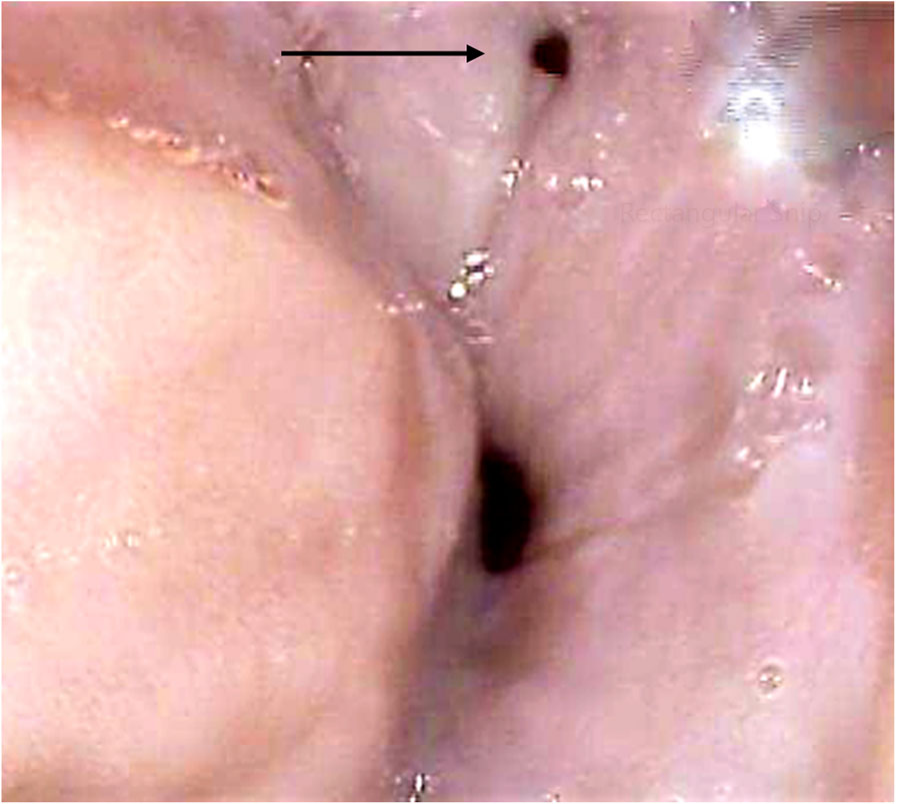
Residual ADF (arrow) following treatment for oesophageal lymphoma

**Fig. 3:**
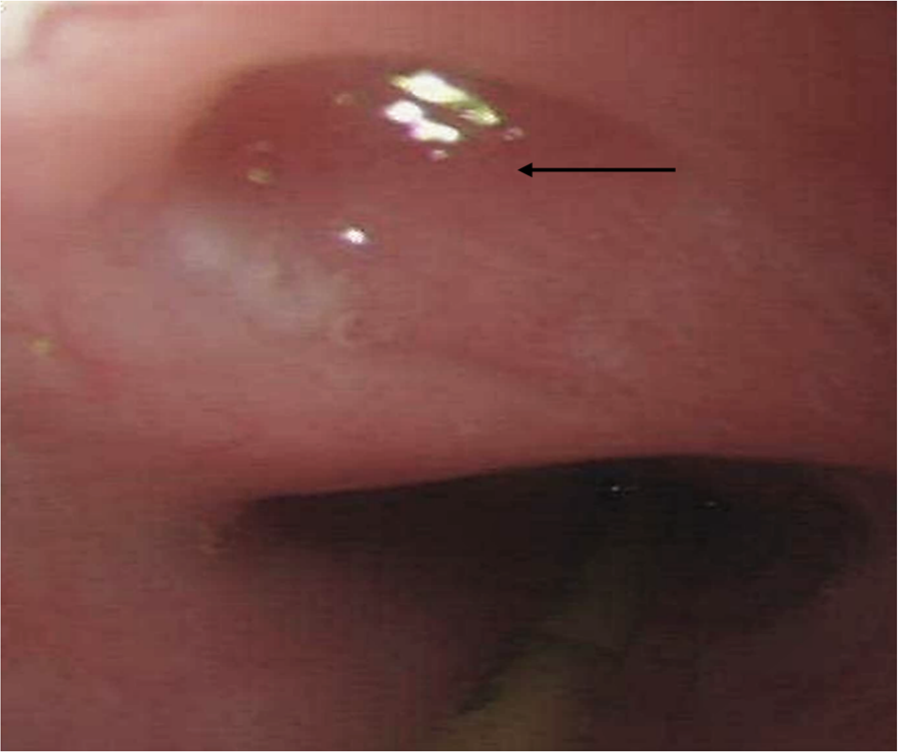
ADF (arrow) in a proximal location, as seen by oesophagoscopy

The two patients with an unknown cause of fistula had a very long history of symptoms, and had been managed in the community with a diagnosis of asthma (Table 2). The median time to ADF development for the three patients with a malignancy was 15 months (range 3–21), with the shortest time affecting a patient who had an oesophageal lymphoma (Patient 7). She had a complete response to chemotherapy, with a residual fistula persisting (Fig. 2). Both patients with TB had a long interval after curative medical therapy, although they had suggestive symptoms for some time prior to referral. Most patients presented with recurrent chest infections and symptoms suggestive of aspiration. Of these, one patient (4) presented with acute respiratory failure owing to overwhelming infection caused by aspiration. Of interest, he had a fistula affecting the very proximal trachea (Fig. 3).

**Table 2:** Anatomical and Clinical Features of the ADFs

Patient	Time to ADF Development (months)	Fistula Site	Fistula Size (mm)	Main Symptoms	Endotherapy to Treat ADF
1	15	Proximal trachea 20 cm	12	Aspiration	×3 stents
2	> 30 years	Oesophagus-right bronchus intermedius 30 cm	12	Recurrent chest infections	–
3	21	Gastric conduit-lung 25 cm	16	Recurrent chest infections	Endoclip
4	> 30 years	Proximal trachea 17 cm	5	Aspiration; Respiratory embarrassment	–
5	144	Distal oesophagus-lung 38 cm	15	Haemoptysis	–
6	3	Oesophagus-carina 24 cm	15	Recurrent chest infections	×3 stents
7	3	Oesophagus- left main bronchus 26 cm	5	Recurrent chest infections	–
8	> 20 years	Proximal trachea 17 cm	3	Recurrent chest infections	–

**Fig. 4:**
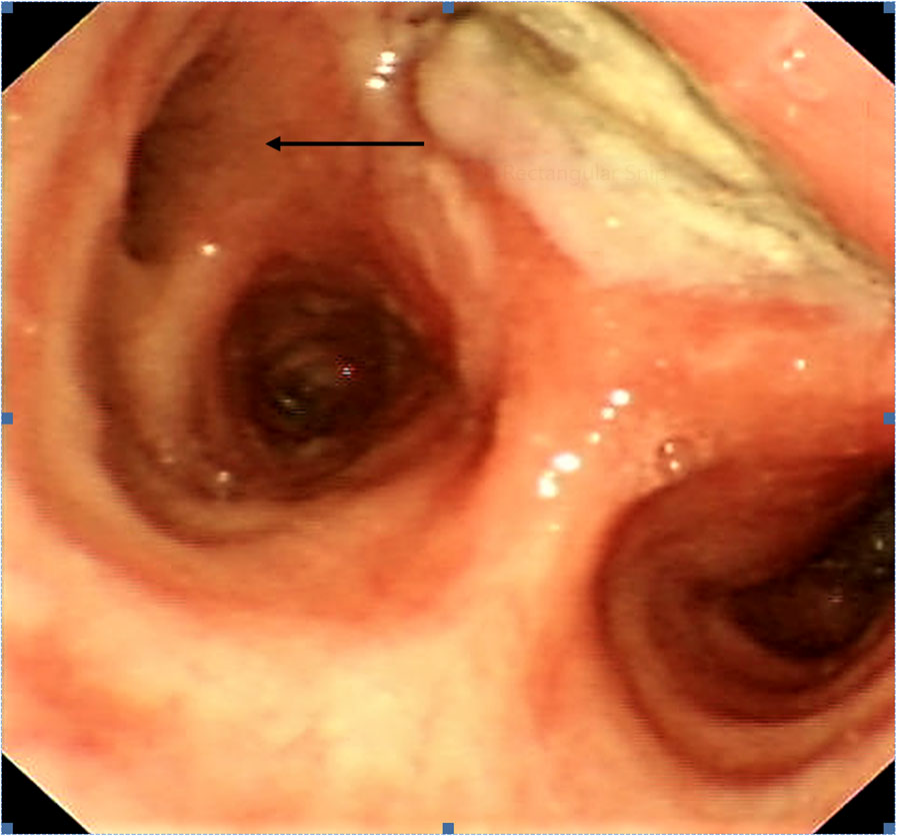
A bronchoscopic image of the ADF (arrow) close to the carina

The size of the fistula ranged from 3 to 16 mm, with the larger defects affecting those who had a prior diagnosis of carcinoma or who underwent surgical treatment. The location of the fistula in relation to the airway too was variable, reflecting the site of underlying disease. Those thought to be congenital were very proximal. Those secondary to TB were both distal, involving the smaller bronchi and lung parenchyma at the original Ghon focus. Patient 6 presented with Boerhaave’s Syndrome, and was initially managed with surgery to repair the oesophageal defect. However, he subsequently re-leaked, which again was managed surgically with a repair over a T-tube, but then developed a stricture at the site of injury, which was treated with an oesophageal stent. This eroded into the airway at its proximal extent, causing a fistula at 24 cm, with subsequent referral to our unit (Fig. 4).

After the diagnosis of ADF, three patients underwent further endotherapy in an attempt to manage the fistula prior to referral to our unit. Patient 1 had 3 oesophageal stents placed, but given the proximal location of the ADF, these all slipped distally. Patient 3, who had developed an ADF between the airway and a gastric conduit, most likely after a sub-clinical leak, had endoclips placed via flexible gastroscopy which failed to close the ADF. Patient 6 too had a stent^s^ placed to treat the fistula without the desired effect.

### Surgical intervention for ADF treatment

Three patients (3, 4, 8) underwent a primary repair of their ADF. Patient 3 required a thoracotomy given that the ADF was communicating with a gastric conduit and Patients 4 and 8 had proximally-sited fistulae approached through the neck. For the former case, an intercostal flap was interposed between the suture lines and for the latter two, the strap muscles were similarly utilised. These fistulae were small and the quality of tissue was sufficiently good to enable primary repair. Patient 4 presented as an acute emergency following aspiration -intubated- and a laparotomy was performed prior to repair, in order to place a feeding jejunostomy and venting gastrostomy (Table 3).

**Table 3:** Surgical Intervention for the Correction of ADF, and Post-Operative Complications

Patient	Operation	Incision	Phases	Reconstruction	Complications
1	Oesophageal Exclusion and fistula repair with strap muscle	Left collar Right PL^a^ thoracotomy Laparotomy	2 phase	Retrosternal Gastric conduit	–
2	Fistula Repair with intercostal muscle	Right PL^a^ thoracotomy	1 phase	n/a	Respiratory infection
3	Fistula Repair with intercostal muscle	Right PL^a^ thoracotomy	1 phase	n/a	–
4	Fistula Repair with strap muscle	Bilateral collar Laparotomy	1 phase	n/a	Respiratory failure RIP
5	Right lower lobe resection and fistula repair	Right PL^a^ mini-thoracotomy	1 phase	n/a	–
6	Oesophageal Exclusion and fistula repair	Left collar Right PL^a^ thoracotomy Laparotomy	2 phase	Retrosternal Colonic conduit	–
7	Oesophagectomy and fistula repair with intercostal muscle	Left collar Right PL^a^ thoracotomy Laparotomy	1 phase	Retrosternal Gastric conduit	Anastomotic leak
8	Fistula Repair with strap muscle	Bilateral collar	1 phase	n/a	–

^a^PL- Postero-lateral

Two patients (1 and 6) underwent an oesophageal exclusion operation. These were both performed as staged procedures with delayed reconstruction. The reason for performing this operation was that in both patients there was sufficient concern regarding the state of tissue. Patient 1 had previous radiotherapy for an oesophageal SCC, on a background of achalasia requiring a myotomy via thoracotomy several years previously. The degree of tissue inflammation, scarring and adhesions precluded a transthoracic resection. Thus, through a neck incision, the oesophagus was transected above the fistula and an oesophagostomy fashioned with a synchronous repair of the fistula. Distally, the oesophagus and OGJ was transected and a venting gastrostomy fashioned. After a period of optimisation and treatment of longstanding respiratory disease, the patient underwent reconstruction utilising a gastric conduit through the retrosternal space. Patient 6 too had severe inflammation and adhesions in the chest following his surgical management of Boerhaave’s syndrome. Exclusion and subsequent reconstruction with a colonic conduit (he had previously undergone a distal gastrectomy for benign ulcer disease) was performed. In both cases, the native tissues were poor enough to carry a high risk of leak with primary anastomosis at index surgery.

Patient 2 had a distal ADF, approached through a thoracotomy. The right bronchus intermedius was involved, and the ADF and affected parenchyma was excised as a segmentectomy, with oesophageal repair. Patient 5 underwent an exploration of the fistula through a thoractomy with a lobectomy. The fistula, along with associated necrotic parenchyma was excised *en bloc*, with a subsequent suture repair of the oesophagus. Patient 7, after chemotherapy for lymphoma, underwent an oesophageal resection. Again, severe residual inflammation was noted at the time of the surgery precluding repair of a small (5 mm) fistula. In addition, our oncology colleagues felt there was a reasonable risk of future recurrence of lymphoma, thus a resection was advocated. A primary repair of the left main bronchus was performed, utilising a flap muscle graft. A single-phase operation was performed as the anastomosis was at a distinct site to the fistula.

### Morbidity and mortality

Three patients suffered from complications following surgery. Patient 2 developed a severe respiratory infection on Day 4 following surgery, requiring bronchoscopic washout. Patient 7 suffered a small anastomotic leak, necessitating prolonged nil oral alimentation. Her nutrition was maintained with jejunal feeding, and a contrast swallow at 7 weeks confirmed complete healing of the leak, after which gradual oral feeding was commenced.

Patient 4, who presented with respiratory failure, was affected by severe post-operative recurrent chest infections, and an inability to wean off mechanical ventilation. This had been anticipated, and hence a tracheostomy had been placed at the time of surgery. Despite 4 months of intensive management, he passed away with respiratory failure and multiple organ dysfunction. He represents the only mortality in this series.

Normal oral alimentation following surgery was achieved in all patients, bar patient 4. The time to attain this milestone ranged from 2 to 3 weeks in all cases, with the exception of patient 7 who had suffered a leak. She required supplemental enteral feeding at home for a short period. We reserve the use of contrast studies and formal swallow assessment for the very proximal fistulae, where the risk of leak and aspiration is highest. At the time of last follow-up, no patient demonstrated clinical evidence of recurrent ADF.

### Hospital episodes statistics (HES)

We performed a search of the English national HES database to assess the reported incidence of ADFs in the UK between 2000 and 2012 (Table 4). Only 71 cases were found. However, we noted that the terms used in the HES system to record an episode or event related to an ADF were difficult to identify and, we suspect, many patients with an ADF were not coded correctly and thus not recorded. Of the 71 cases, 17 (23.9%) underwent documented treatment: 9 (12.7%) were treated surgically and 8 (11.3%) underwent oesophageal stent placement. Most patients (56.4%) presented with respiratory symptoms. The 30-day and 90-day mortality rates were 32.4 and 42.3% respectively, although for cases managed in high-volumes centres this fell to 25 and 31.3% respectively.

**Table 4:** HES data search for ADFs between 2000 and 2012 (^a^ denotes hospitals that perform ≥20 oesophageal cancer resections per year)

HES data (2000–2012)	*n*	%	*p*
Age ≥ 70 years	35	49.3	
Sex			
Female	35	49.3	
Male	36	50.7	
Treatment	17	24	
Surgery	9	12.7	
Stenting of oesophagus	8	11.3	
Unknown	54	76	
Presenting Clinical Feature			
Pneumonia	18	25.4	
Pleural effusion	22	31	
Pulmonary embolus	1	1.4	
Ischaemic cardiac event	1	1.4	
Unknown	29	40.8	
All Hospitals			
30-day mortality	23	32.4	
90-day mortality	30	42.3	
Specialist Centres^a^	16	22.5	
30-day mortality	4	25	0.473
90-day mortality	5	31.3	0.311

## Discussion

This series demonstrates the techniques, challenges and strategies utilised in the surgical management of ADFs. The key aspect in the approach to such intervention is the multi-disciplinary nature of care, utilising the experience of several distinct surgical specialities. Pre-operative respiratory optimisation should be aggressive, and ideally patients should be weaned off artificial ventilation prior to surgery [2, 3]. As the HES data demonstrates, apart from being rare, there is a deficiency in accurate diagnosis, coding and documentation of this condition.

The range of operations that can be utilised reflect the nature of underlying disease [1–4]. The determinants of which operation will be performed are mainly the site and size of the ADF, and the state of the affected tissue- itself a reflection of the preceding disease and treatment. For the most proximal ADFs, an approach through a neck incision is the most desirable. A pivotal stratagem here involves mobilising the oesophagus circumferentially. This manoeuvre allows the more distal structures to be brought superiorly into the wound, making further surgery easier and away from the rigid confines of the thoracic inlet. If necessary, the medial clavicle and sternoclavicular joint can be excised, a procedure which does not cause future disability [7]. Distal ADFs necessitate a thoracotomy. These ADFs can cause significant damage to the lung parenchyma, affecting the compliance by causing fibrosis [8]. Thus, where necessary, we advocate a lobar or segmental resection, with closure of the associated distal bronchi. Where an oesophagectomy is indicated, we favour leaving a cuff of oesophageal tissue around the trachea. This enables a dissection plane away from the airway, and the remnant tissue can be incorporated into the repair. This manoeuvre also lessens the future risk of tracheal stenosis [3].

Although not universally favoured, we have found the oesophageal exclusion operation a beneficial option in specific patients. Gross contamination of the airway, or in patients who have had a previous leak or radiotherapy, results in significant inflammation and adhesions in the thorax. Attempting a resection in this circumstance is hazardous, and if there is no definite indication to resect, the oesophagus can be safely left in situ. It is important that a generous venting gastrostomy is sited, from where the oesophagus can be accessed. We have successfully performed a retrograde endoscopy through this, and administered therapeutic agents required for treatment of disuse oesophagitis. Most importantly, it enables venting of oesophageal mucous. Some authors favour a single operation rather than a staged approach for resection or repair and reconstruction [3, 7]. In our experience, fashioning an anastomosis in a contaminated field increases the chance of a leak. In such an event, the fistula has a high chance of recurrence. Most importantly, however, a significant leak may necessitate a far more morbid operative intervention. Indeed, any resultant fistula is likely to be more difficult to treat, if this remains at all possible.

Several other series also demonstrate the complexity of surgical intervention [1–4, 9]. A feature of these reports is that experience is limited to a few specialist units where an expertise in ADF management is present. This results in better outcomes, and facilitates an environment where management is continually improved. In our series, there was one post-operative death. The remaining patients all achieved normal alimentation soon after surgery, with no evidence of ADF recurrence at a median of 32 months. These results are comparable to other dedicated units. Mathisen et al demonstrated a mortality rate of 10.5% in a series of 38 patients, many of whom underwent tracheal reconstruction, with excellent long-term outcomes [3]. In a subsequent report, highlighting 35 years’ experience in the management of ADFs, the operative mortality rate fell to 2.8%, reflecting the effect of concentrating cases in specialist units [1]. Shen et al similarly report a low mortality rate of 5.7%, with a post-operative complication rate of 54.3%, and an oesophageal leak rate of 11.4% [9]. Baisi et al reported on 31 patients, of whom 26 underwent simple closure of the oesophageal and tracheal defects. Operative mortality was 3.2%, with a recurrence rate of 6.4% [10].

Non-operative techniques can be used to manage ADFs. Oesophageal stenting is the most common intervention, and although it plays a key role in some patients, they can themselves *cause* fistulae and may affect future surgical intervention [11, 12]. Newer endoscopic techniques utilising endoscopic suturing, clip placement or tissue glue, may have an increasing role in the management of ADFs in the future. Thus, early referral to a dedicated unit is advocated, as the *whole* management of the patient can be pursued where the complete skill-set, including access to novel treatments, is present. By focusing care in specific units, the expertise in all facets of management and outcomes can continually improve.

## Conclusion

In summary, for a select group of patients an operative approach can be a truly life-saving intervention. Although surgery is not without risk, it offers the best chance of cure of ADF with a very low risk of recurrence and a return to normal oral alimentation.

## Abbreviations

ADF: Aerodigestive Fistula
HES: Hospital Episodes Statistics
MDT: Multidisciplinary Team
OGJ: Oesophago-Gastric Junction
TB: Tuberculosis
TOF: Tracheo-Oesophageal Fistula

## References

[1] Muniappan A, Wain JC, Cameron D (2013). Surgical treatment of nonmalignant tracheoesophageal fistula: a thirty-five year experience. Ann Thorac Surg.

[2] Macchiarini P, Verhoye J-P, Chapelier A (2000). Evaluation and outcome of different surgical techniques for postintubation tracheoesophageal fistulas. J Thorac Cardio Vasc Surg.

[3] Mathisen DJ, Grillo HC, Wain JC (1991). Management of Acquired Nonmalignant Tracheoesophageal Fistula. Ann Thorac Surg.

[4] Meunier B, Stasik C, Raoul JL (1998). Gastric bypass for malignant esophagotracheal fistula: a series of 21 cases. Eur J Card Thorac Surg.

[5] Grillo HC. Acquired tracheoesophageal fistula and bronchoesophageal. In: Frillo HC, ed. Surgery of the Trachea and Bronchi. New York: BC Dekker Inc., 2003, 341–356.

[6] Bartels HE, Stein HJ, Siewert JR (1998). Tracheobronchial lesions following oesophagectomy: prevalence, predisposing factors and outcome. Br J Surg.

[7] Barkley C, Orringer MB, Iannettoni MD, Yee J (2003). Challenges in reversing esophageal discontinuity operations. Ann Thorac Surg.

[8] Diddee R, Shaw IH (2006). Acquired trachea-oesophageal fistula in adults. BJA: CEACCP.

[9] Shen KR, Allen MS, Cassivi SD (2010). Surgical management of acquired nonmalignant tracheoesophageal and Bronchoesophgageal fistulae. Ann Thorac Surg.

[10] Baisi A, Bonavina L, Narne S (1999). Benign trachea-esophageal fistula: results of surgical therapy. Dis Esoph.

[11] Desiree van den Bongard HJ, Boot H, Baas P, Taal BG (2002). The role of parallel stent insertion in patients with esophagorespiratory fistulas. Gastointest Endosc.

[12] Ellul JP, Morgan R, Gold D (1996). Parallel self-expanding covered metal stents in the trachea and oesophagus for the palliation of complex high tracheo-oesophageal fistula. Br J Surg.

